# T-cell exhaustion in the tumor microenvironment

**DOI:** 10.1038/cddis.2015.162

**Published:** 2015-06-18

**Authors:** Y Jiang, Y Li, B Zhu

**Affiliations:** 1 Institute of Cancer, Xinqiao Hospital, Third Military Medical University, Chongqing, China; 2 Center for Experimental Therapeutics and Reperfusion Injury, Perioperative and Pain Medicine, Harvard Institutes of Medicine, Brigham and Women's Hospital and Harvard Medical School, Boston, MA, USA

## Abstract

T-cell exhaustion was originally identified during chronic infection in mice, and was subsequently observed in humans with cancer. The exhausted T cells in the tumor microenvironment show overexpressed inhibitory receptors, decreased effector cytokine production and cytolytic activity, leading to the failure of cancer elimination. Restoring exhausted T cells represents an inspiring strategy for cancer treatment, which has yielded promising results and become a significant breakthrough in the cancer immunotherapy. In this review, we overview the updated understanding on the exhausted T cells in cancer and their potential regulatory mechanisms and discuss current therapeutic interventions targeting exhausted T cells in clinical trials.

## Facts

- T-cell exhaustion is a hyporesponsive state of T cells in chronic environment, with increased inhibitory receptors, decreased effector cytokines and impaired cytotoxicity.
- Most T cells in tumor microenvironment are exhausted, leading to cancer immune evasion.
- PD-1 is the major inhibitory receptor regulating T-cell exhaustion, T cells with high PD-1 expression lose the ability to eliminate cancer.
- Reversing T-cell exhaustion represents an inspiring strategy to treat cancer.

## Open Questions

- What is the definition of ‘exhausted T cell'?
- What is the differentiation process of T cells in tumor microenvironment?
- How does tumor microenvironment regulate T-cell exhaustion?
- Reversing T-cell exhaustion represents promising cancer therapy, what are the limitations and adverse reactions? How to improve treatment efficiency?
- What should be further studied about T-cell exhaustion?
- What are the similarities and differences between T-cell exhaustion in chronic infection and T-cell exhaustion in cancer?

T cells are the major force of adaptive immunity. Following exposure to foreign antigens, naive T cells (CD44^low^CD62L^hi^) activate and expand greatly during the first 1–2 weeks. Subsequently, T cells acquire effector functions, including the production of effector cytokines and granzyme/perforin-mediated cytotoxicity. After the peak of T-cell proliferation, 90–95% of effector T cells (CD44^hi^CD62^low^) die via apoptosis. The surviving T cells differentiate into memory T cells and are maintained in the resting state.^1^ The memory T-cell differentiation is observed in most cases of acute inflammation.^2^ Upon re-exposure to the same or similar antigens, memory T cells expand more quickly and regain higher effector function than naive T cells.^3, 4^ These capacities allow memory T cells to persist and to confer protective immunity for a long time, even after the antigen withdraws.

In contrast, tumor antigens are weakly immunogenic self-molecules, and most tumor-specific T cells are of low precursor frequencies and low T-cell receptor (TCR) affinity because tumor-specific T cells with high avidity are deleted during the thymic selection process.^2^ In addition, the process of antigen presentation is impaired in tumor microenvironment (TME), leading to insufficient priming and boosting of T cells.^5^ Although effector T cells enter TME, they are regulated by a complex immunosuppressive network that consists of cancer cells, inflammatory cells, stromal cells and cytokines. Among these TME components, cancer cells, inflammatory cells and suppressive cytokines have crucial roles in regulating T-cell phenotype and function. These components drive T cells terminally to differentiate into ‘exhausted' T cells.^5^

**Figure 1:**
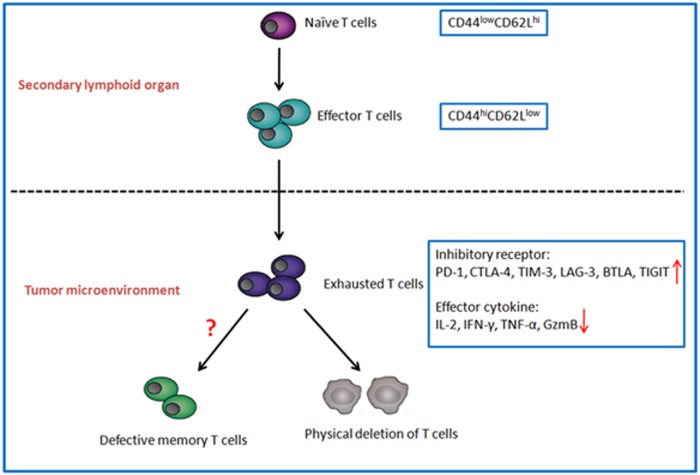
T-cell exhaustion and differentiation in TME. Naive T cells (CD44^low^CD62L^hi^) activate and differentiate into effector T cells (CD44^hi^CD62L^low^) in secondary lymphoid organ. When effector T cells enter TME, they are polarized into exhausted T cells, with decrease in effector cytokines (IL-2/IFN-*γ*/TNF-*α*/GzmB) and increase in inhibitory receptors (PD-1/CTLA-4/TIM-3/LAG-3//BTLA/TIGIT). Subsequently exhausted T cells may turn to be defective memory T cells or be deleted physically

Exhausted T cells were primarily identified in a chronic lymphocytic choriomeningitis virus (LCMV) infection model. The LCMV-specific CD8^+^T cells expressing activation markers (CD69^hi^CD44^hi^CD62L^low^) were unable to perform the anti-viral functions.^6^ T-cell exhaustion is a state of T-cell dysfunction in chronic environment, exhausted T cells express high levels of inhibitory receptors, including programmed cell death protein 1 (PD-1), lymphocyte activation gene 3 protein (LAG-3), T-cell immunoglobulin domain and mucin domain protein 3 (TIM-3), cytotoxic T lymphocyte antigen-4 (CTLA-4), band T lymphocyte attenuator (BTLA) and T-cell immunoglobulin and immunoreceptor tyrosine-based inhibitory motif domain (TIGIT).^7, 8, 9, 10, 11, 12^ The other principal characteristic of exhausted T cells is the loss of function in a hierarchical manner. Such functions as interleukin-2 (IL-2) production and *ex vivo* killing capacity are lost at the early stage of exhaustion,^13^ whereas tumor necrosis factor-*α* (TNF-*α*) production is lost at the intermediate stage, interferon-*γ* (IFN-*γ*) and granzyme B (GzmB) production are lost at the advanced stage of exhaustion.^14^ The first evidence connecting exhausted T cells with TME was that overexpressed programmed cell death 1 ligand 1 (PD-L1, the ligand of PD-1) on mouse mastocytoma P815 cells rendered them less susceptible to the specific TCR-mediated lysis by cytotoxic T cells *in vitro* and remarkably enhanced their tumorigenesis and invasiveness *in vivo*, which indicates that the expression of PD-L1 contributes to immune evasion.^15^ Most T cells in TME differentiate into exhausted T cells, express high levels of inhibitory receptors and produce less effector cytokines, and lose the ability to eliminate cancer. T-cell exhaustion may be related with defective memory T cells formation, and the final stage of T-cell exhaustion is the physical deletion, by which severely exhausted T cells are cleared in TME (Figure 1).

In this review, we discuss the potential mechanisms involved in T-cell exhaustion in TME. We also introduce the therapeutic interventions that target exhausted T cells in clinical trials.

## Intrinsic Mechanisms Involving in T-Cell Exhaustion and Differentiation in TME

Complete activation of T cells requires three signals, the first signal is the interaction of antigenic peptide–MHC complex with TCR, the second signal is costimulatory or co-inhibitory signal provided by antigen-presenting cells, the third signal is the stimulation by extracellular cytokines such as IL-2.^16^ Among these signals, the second signal determines the promotion or inhibition of T-cell cytokine production and effector function, appropriate co-inhibitory signals dampen inflammation to avoid tissue damage from excessive immune reaction, whereas durative and overmuch co-inhibitory signals lead to T-cell hyporesponsiveness.^17^ Co-inhibitory signals are primarily mediated by inhibitory receptors that are the major phenotypes of exhausted T cells.^18^ Genomic studies on exhausted T cells in chronic LCMV infection defined specific molecular pathways distinct from effector T cells and memory T cells, primarily including increased inhibitory receptors and decreased cytokine signaling pathways, and so on.^19^ Consistent with chronic infection, T cells in TME also exhibit exhausted phenotype and function. Exhausted T cells in cancer express high levels of inhibitory receptors, including PD-1, CTLA-4, TIM-3, LAG-3, BTLA and TIGIT, as well as show impaired effector cytokine production, such as IL-2, TNF-*α*, IFN-*γ* and GzmB (Figure 1).

PD-1 expression was markedly upregulated on tumor-infiltrating CD8^+^ T cells and correlated with reduced cytokines in Hodgkin's lymphoma, melanoma, hepatocellular carcinoma and gastric cancer patients.^20, 21, 22, 23, 24^ PD-1 expression on Jurkat cells increased after co-cultured with cancer cells, blockade of PD-1 pathway successfully restored T-cell function.^25^ CTLA-4 is an immune checkpoint receptor expressed only on T cells, and it competes with the costimulatory molecule CD28 in binding the ligands CD80/CD86 and initiating intracellular inhibitory signals.^26^ The interaction of CTLA-4 with CD80/CD86 generates inhibitory effects on T-cell activation and IL-2 production.^27^ One-third to half of CD8^+^ tumor-infiltrating lymphocytes (TILs) co-expressed PD-1 and CTLA-4, PD-1^+^CTLA-4^+^CD8^+^TILs were more severely exhausted in proliferation and cytokine production, whereas dual blockade of PD-1 and CTLA-4 enhanced T-cell function in cancer.^28^ Both PD-1 and CTLA-4 inhibited the activity of Akt, a crucial molecular in regulating glucose metabolism of T cells by elevating glucose transporter 1 expression and glycolysis, suggesting that glucose metabolism may contribute to T-cell exhaustion.^29^ In addition, TIM-3, LAG-3, BTLA and TIGIT also regulate T-cell exhaustion in cancer, which has been demonstrated in both animal experiments and cancer patients below.

In tumor-bearing animal models, co-expression of PD-1/TIM-3 was generally observed on TILs, among these cells, TIM-3^+^PD-1^+^CD8^+^TILs represented the predominant subset and exhibited greater exhausted phenotypes than TIM-3^-^PD-1^-^ and TIM-3^+^PD-1^-^CD8^+^TILs, as defined by failure to proliferate and produce IL-2, TNF-*α* and IFN-*γ*, dual blockade of PD-1 and TIM-3 restored the anti-tumor function of exhausted CD8^+^T cells.^30^ In melanoma patients, TIM-3^+^PD-1^+^tumor-specific CD8^+^T cells were more dysfunctional than TIM-3^-^PD-1^+^ and TIM-3^-^PD-1^-^T cells, producing less IFN-*γ*, TNF-*α* and IL-2.^31^ Co-expression of PD-1/LAG-3 was also observed on CD8^+^TILs in tumor-bearing animal models, PD-1^+^LAG-3^+^TILs exhibited more exhausted phenotype and function than single positive or negative TILs, dual blockade of PD-1 and LAG-3 resulted in tumor regression.^32^ Similarly, LAG-3^+^PD-1^+^CD8^+^T cells were more dysfunctional in IFN-*γ* and TNF-*α* production compared with LAG-3^+^PD-1^-^ or LAG-3^-^PD-1^-^CD8^+^subsets in human ovarian cancer.^33^ BTLA mediated the functional inhibition of CD8^+^T cells by its ligand herpes virus entry mediator,^34^ BTLA^+^PD-1^+^TIM-3^+^CD8^+^T cells were most dysfunctional among NY-ESO-1-specific CD8^+^T cells in melanoma patients. Combined blockade of BTLA, PD-1 and TIM-3 enhanced the proliferation and function of tumor-specific CD8^+^T cells, suggesting a role for BTLA in regulating T-cell exhaustion in advanced melanoma.^11^ Recently, another co-inhibitory receptor TIGIT was demonstrated in T-cell exhaustion. Similar with CTLA-4/CD28 and CD80/CD86, TIGIT competes with CD226 in binding the same set of ligands CD115/CD112, CD226 is a costimulatory signal for T-cell response, whereas TIGIT is a negative regulator.^12, 35^ Tumor antigen-specific CD8^+^T cells and CD8^+^TILs from melanoma patients expressed high levels of TIGIT, and TIGIT^+^CD8^+^T cells also co-expressed PD-1, combined blockade TIGIT with PD-1 promoted CD8^+^T cell proliferation and cytokine production.^36^ In tumor-bearing mouse, TIGIT was also highly expressed on TILs, antibody co-blockade of TIGIT and PD-L1 synergistically and specifically boosted CD8^+^T cell effector function, resulting remarkable tumor clearance.^37^ Above results suggest that PD-1 is the major regulator of T-cell exhaustion, in addition, the pattern of inhibitory receptor co-expression on the same CD8^+^T cell probably determines the severity of T-cell exhaustion, combined blockade inhibitory receptors represents inspiring strategies for cancer therapy.

**Figure 2:**
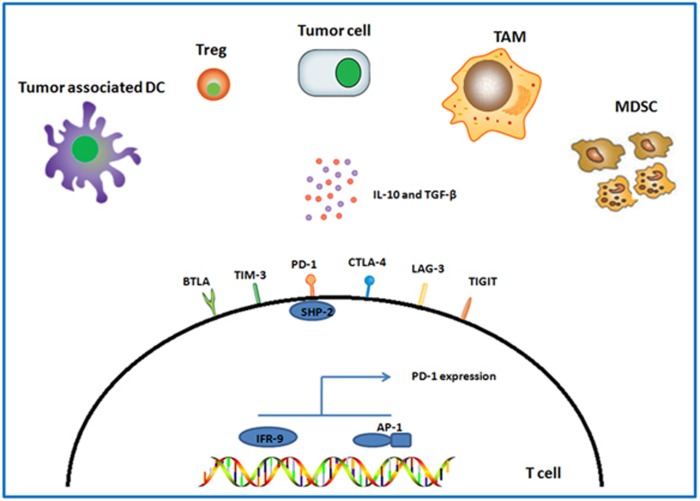
Potential regulatory mechanisms of T-cell exhaustion in TME. Cancer cells and stromal cells (tumor-associated DC, Treg, TAM and MDSC) are major extrinsic cells that regulate T-cell exhaustion, and IL-10 and TGF-*β* are both important extrinsic cytokines involved in exhausted process of T cells. Inhibitory receptors PD-1, CTLA-4, Tim-3, BTLA, LAG-3 and TIGIT on T cells are the major intrinsic regulatory factors of T-cell exhaustion. SHP-2 is the downstream of PD-1, IRF-9 and AP-1, which regulate PD-1 expression in transcriptional level

The transcription factors Blimp-1, T-bet, NFATc1 and BATF are critical for T-cell exhaustion in chronic infection,^38, 39, 40, 41^ but the intracellular signal pathway involved in regulation of exhausted T cells in cancer remains poorly understood. PD-1 was elevated markedly in tumor-infiltrating T cells of Hodgkin's lymphoma. Blockade of the PD-1 signaling pathway inhibited the phosphorylation of SHP-2, a SH2-containing tyrosine-specific protein phosphatase, and restored the IFN-*γ*-producing function, indicating that SHP-2 phosphorylation is involved in the PD-1 downstream intracellular signal transduction (Figure 2).^20^

In addition, IFN-*α* induces and maintains PD-1 expression on the TCR-engaged primary mouse T cells through the association between IFN-responsive factor 9 and the IFN stimulation response element, suggesting the role of IFN-responsive factor 9 on regulating T-cell exhaustion.^42^ The tumor-infiltrating T cells exhibit upregulated expression of activator protein 1 (AP-1) subunit c-Fos. The ectopic expression of c-Fos in T cells promote tumor progression by inducing PD-1 expression via direct binding to the AP-1-binding site in the Pdcd1 (gene encoding PD-1) promoter, indicating that c-Fos directly regulates T-cell exhaustion in cancer (Figure 2).^43^

Exhausted T cells express low levels of CD122 (the *β*-chain of the IL-2 and IL-15 receptor) and CD127 (the IL-7 receptor *α*-chain) in chronic infection so that they lose the ability to survive long-term without antigens via IL-7- and IL-15-mediated memory maintenance.^13^ Although T cells with exhausted phenotype transferred into naive mice regained the ability to proliferate and control viral infection,^44^ it was also demonstrated that exhausted T cells hardly recovered normal differentiation of memory T cells in antigen-free recipients in infection models.^45^ These findings suggest that simply removing the antigen cannot restart normal memory T-cell differentiation. The effects of T-cell exhaustion on memory T-cell formation have also been demonstrated in TME. In tumor-bearing mouse models, TILs are divided into three subsets, namely, PD-1^+^TIM-3^+^, PD-1^-^TIM-3^+^ and PD-1^-^TIM-3^-^ T cells. TIM-3^+^PD-1^+^T cells contain the largest population of effector/memory T cells with a high expression of CD44 and low expression of CD62L, but consist of the lowest population of central memory (CD44^hi^CD62L^hi^) cells. The majority of the three TIL subsets express low to intermediate levels of CD44, whereas the CD44^int^ cells are the lowest in TIM-3^+^PD-1^+^T cells.^30^ Owing to these results, we speculate that exhausted T cells in TME favor the differentiation of effector/memory T cells instead of central memory T cells, through which the long-term maintenance of anti-tumor immunity is impaired (Figure 1).

T-cell exhaustion is related to physical T-cell deletion in cancer. PD-L1 is highly expressed in various tumor tissues, and the expression of PD-L1 inversely correlates with prognosis and survival. PD-L1-associated T-cell apoptosis is one of several potential mechanisms, which is supported by the inverse correlation between PD-L1 expression in tumor tissues and the number of TILs.^46^ In hepatocellular carcinoma patients, immunohistochemical staining indicated that PD-L1 expressed hepatoma cells and apoptotic infiltrating CD8^+^T cells were both enriched in tumor sections. IFN-*γ* secreted by CD8^+^T cells induced PD-L1 expression on hepatoma cells, which in turn promoted CD8^+^T cell apoptosis *in vitro.*^47^ Hepatic stellate cells isolated from hematocellular carcinoma expressed high levels of PD-L1, which was associated with enhanced T-cell apoptosis.^48^ These findings suggest that the advanced stage of T-cell exhaustion is the physical deletion (Figure 1).

It is worth mentioning that PD-1/TIM-3 or PD-1/LAG-3 co-expression on CD4^+^ TILs were also observed in a recurrent mouse melanoma model.^30, 32^ These tumor-specific CD4^+^T cells expressed inhibitory receptors, such as PD-1, TIM-3 and LAG-3.^49^ However, whether the function of tumor-infiltrating CD4^+^T cells is decreased or whether these cells are also exhausted remains unknown.

## Extrinsic Mechanisms Regulating T-Cell Exhaustion in Cancer

TME consists of cancer cells, inflammatory cells, stromal cells and cytokines, these components form a complicated immunosuppressive network in cancer, which limits T-cell activation and induces T-cell dysfunction. The potential extrinsic factors involved in T-cell exhaustion in cancer include tumor cells, inflammatory cells and immunosuppressive cytokines (Figure 2).

TME is abundant with tumor antigens. Chronic tumor antigens induce durative activation of T cells in TME, which probably contribute to T-cell exhaustion. The expression of PD-L1 and programmed cell death 1 ligand 2 (PD-L2) is correlated with prognosis in some human malignancies^50, 51, 52, 53, 54, 55, 56, 57, 58, 59, 60, 61, 62^ (Table 1). The PD-L1/PD-1 signaling pathway is a crucial regulatory pathway of T-cell exhaustion in cancer. PD-L1 is abundantly expressed in cancer cells and stromal cells, and blockade of PD-L1/PD-1 enhances T-cell anti-tumor function.^63^ PD-L2 also binds to PD-1 and regulates T-cell function. The constitutive basal expression of PD-L2 is low, but PD-L2 expression can be induced on dendritic cells (DCs), macrophages and mast cells in response to IL-4 and IFN.^64^

Regulatory T (Treg) cells are an inhibitory subset of CD4^+^ T cells that maintain peripheral tolerance and prevent autoimmune diseases. Tregs also accumulate in tumor tissues and the peripheral blood of cancer patients and contribute to immune evasion.^65, 66^ The ectoenzymes CD39 and CD73 on Treg cells have been demonstrated to mediate the generation of pericellular adenosine, which suppressed the function of effector T cells by activating the adenosine A2A receptor.^67, 68^ High expression of CD25 on Tregs consumed excessive local IL-2, thereby impaired T-cell function.^69, 70^ In addition, inhibitory cytokines derived from Tregs, such as IL-10 and transforming growth factor *β* (TGF-*β*), also suppress the function of effector T cells.^71, 72^

DCs are a subset of professional antigen-presenting cells. Plasmacytoid DCs generated during cancer development can induce Treg differentiation via indoleamine 2,3-dioxygenase (IDO). The increased Tregs secreted IL-10 and significantly upregulated PD-L1 on conventional DCs.^73^ In the transgenic adenocarcinoma of the mouse prostate model, a population of DC with plasmacytoid phenotype was observed in TME. These tumor-associated DCs expressed low levels of the costimulatory ligands CD80, CD86 and CD40 but high levels of genes associated with T-cell exhaustion, including PD-L1 and IDO.^74^ These data indicate that plasmacytoid DCs also contribute to T-cell exhaustion.

Macrophages are critical cells in the innate immunity that defend the host against foreign pathogens. They are generally classified into two extreme phenotypes: M1 macrophages produce considerable pro-inflammatory cytokines, whereas M2 macrophages secrete several growth factors that activate the process of tissue repair and suppress adaptive immune responses.^75^ Macrophages accumulated in cancer are termed tumor-associated macrophages (TAMs).^75^ Tumor-derived signals such as M-CSF, CCL2, VEGF and angiopoietin-2 recruit blood monocytes through the tumor vessels and promote the polarization of macrophages in cancer sites.^76, 77^ TAMs exhibit an M2-like phenotype and possess pro-tumor immunity. Therefore, the correlation between TAM density and the patient's prognosis is negative.^78^ The overexpression of CCL2 by murine fibrosarcoma cells resulted in an increase in TAMs numbers, which contributed to tumor growth *in vivo.*^79^ TAMs suppressed T-cell activation and proliferation by producing suppressive mediators, including IL-10 and TGF-*β*. In addition, TAMs were unable to trigger Th1-polarized immune responses rather than induce Treg formation.^80^ The TAMs from renal cell carcinoma patients induced the skewing of autologous blood-derived CD4^+^T cells toward a more exhausted phenotype, with decreased production of effector cytokines and enhanced expression of PD-1 and TIM-3.^81^

The accumulation of myeloid-derived suppressor cells (MDSCs) has been recognized as a major mechanism to promote carcinogenesis. These cells originate from myeloid tissue and are comprised of myeloid cell progenitors, precursors of DCs, monocytes, macrophages and granulocytes.^82^ They are typically CD11b^+^CD33^+^CD34^+^CD14^-^HLA-DR^-^ cells in cancer patients, and are CD11b^+^Gr-1^+^ cells in tumor-bearing mice. MDSCs are further divided into ‘monocytic' (CD11b^+^Ly6G^low^Ly6C^hi^) and ‘granulocytic/neutrophil-like' (CD11b^+^Ly6G^hi^Ly6C^low^) MDSCs.^83^ They are considered to be a population of inhibitory cells because they suppress T-cell activation and induce T-cell exhaustion by multiple mechanisms. For example, in ovarian carcinoma animal models, CD11b^+^Gr-1^+^cells with a high expression of PD-L1 and CD80 markedly inhibited antigen-specific immune responses, whereas blockade of PD-L1 and CD80 in Gr-1^+^CD11b^+^cells abrogated immune suppression.^84, 85^ MDSCs derived from IL-10-stimulated DCs exhibited enhanced PD-L1 expression, and these cells induced T-cell dysfunction via the PD-L1/PD-1 signaling pathway.^86^ These results uncover the role of MDSCs in regulating T-cell exhaustion in cancer.

Immunosuppressive cytokines, such as TGF-*β* and IL-10, are crucial factors during T-cell exhaustion. TGF-*β* in TME is mainly secreted by cancer cells, immune cells and fibroblasts.^87^ The role of the TGF-*β* signaling pathway in cancer is complex and paradoxical, varying by cell type and the stage of cancer. In general, TGF-*β* mediates tumor suppression via the inhibition of cancer cell proliferation and the induction of cancer cell apoptosis in early stages. It also promotes tumor cell invasion and metastasis through the modulation of immune response in later stages.^88^ Recent research has shown that TGF-*β* directly suppresses the cytotoxicity of CTLs by the transcriptional repression of genes encoding key functional cytokines, such as perforin, granzymes and cytotoxins.^72, 89^ Tumor-derived TGF-*β* directly suppressed CTL effector function by elevating miR-23a and downregulating Blimp-1, a key transcription factor involved in T-cell differentiation.^90^ In addition, the naive T cells treated with TGF-*β* favor the differentiation into Treg cells, which are involved in the T-cell exhaustion.^91^

Elevated IL-10 in TME is primarily secreted by TAMs, CD4^+^regulatory T cells and cancer cells.^80, 92^ IL-10 can exert anti-tumor activity through NK-mediated tumor cell lysis induced by downregulation of MHC-I. On the other hand, IL-10 also dampened anti-tumor immunity via an immunosuppressive role on DCs and macrophages.^93^ IL-10 induced PD-L1 expression on DCs, which in turn mediated the exhaustion process of T cells.^86^ Moreover, IL-10 has a significant role in the induction of Tregs.^94^ These findings demonstrate that IL-10 contributes to T-cell exhaustion in TME.

## Therapeutic Interventions by Reversing Exhausted T Cells in Cancer

Research from bench to bedside indicates that the blockade of inhibitory receptors is a great breakthrough in cancer therapy. The blocking antibodies in clinical development primarily include CTLA-4 and PD-1/PD-L1 antibodies. Ipilimumab is an anti-CTLA-4 monoclonal antibody (mAb) approved for melanoma treatment by the US Food and Drug Administration (FDA) in 2011, and it is the first agent to show survival benefits for metastatic melanoma patients.^95^ Ipilimumab has also been evaluated in clinical trials for metastatic prostate cancer and advanced non-small cell lung cancer, ipilimumab alone or combined with other anti-tumor therapies showed significant clinical benefits for cancer patients.^96^ Tremelimumab is another anti-CTLA-4 monoclonal antibody in clinical trials for cancer therapy, and showed anti-tumor activity with a durable response in phases I and II clinical studies^97^ (Table 2).

PD-L1/PD-1 blockade reverses exhausted T cells and restores anti-tumor function. The PD-1 antibodies (pidilizumab, pembrolizumab and nivolumab) and PD-L1 antibodies (BMS-936559, MPDL3280A and MEDI4736) have been subjected to clinical trials.^98^ Pidilizumab is the first PD-1 mAb for clinical trials, significant clinical benefit and durable response was observed in patients with hematologic malignancies who received pidilizumab treatment.^99^ Pembrolizumab is a PD-1-blocking mAb with no cytotoxic activity against T cells and has been used for advanced melanoma patients in a phase I trial, the safety and tumor response were satisfactory.^100^ Nivolumab is another mAb to PD-1, which was first studied clinically in patients with advanced solid tumors, the objective responses for melanoma, renal cell carcinoma (RCC) and non-small cell lung cancer (NSCLC) patients were durable and lasted over 1 year^63, 101^ (Table 2).

A fully human monoclonal PD-L1 antibody BMS-936559 was studied in clinical trials. Durable tumor regression was observed in advanced melanoma, NSCLC, RCC and ovarian cancer patients with BMS-936559 treatment, and the adverse events were tolerated.^102^ MPDL3280A, a humanized PD-L1-blocking mAb, showed impressive anti-tumor effects in metastatic urothelial bladder cancer, the tolerability and efficacy in other advanced solid tumors were also acceptable.^103^ MEDI4736 is a PD-L1 antibody with modified Fc domain, which was studied in phase I or Ib clinical trials for advanced solid tumors, the results are encouraging^104^ (Table 2).

The combined therapies with immune checkpoint antibodies are also in clinical trials. For example, pidilizumab combined therapy with rituximab was used for follicular lymphoma, pembrolizumab followed by ipilimumab or BRAF inhibitor treatment was used for malignant melanoma patients.^64^ Combined therapy with Nivolumab and ipilimumab was recently applied in melanoma. The rate of objective response rate in malanoma patients received combined therapy was 61%, whereas that in melanoma patients received ipilimumab monotherapy was only 11%, the complete responses in combination group and ipilimumab monotherapy were 22 and 0% separately.^105^ These combination immunotherapies exhibit promising effects in improving outcomes for advanced cancer patients.

## Conclusions and Perspectives

The presence of T cells in TME correlates with favorable prognosis. However, T cells in TME are always in hyporesponsive state. There are several hurdles that limit T cells to eliminate tumors. The major stumbling block for T-cell hyporesponsiveness in cancer is T-cell exhaustion. Exhausted T cells have a unique molecular signature that is markedly distinct from naive, effector or memory T cells. They are defined as T cells with decreased cytokine expression and effector function. Reversing exhausted T cells and restoring anti-tumor potential represents an inspiring strategy to treat cancer. An attractive option of reversing exhausted T cells is to block inhibitory receptors. In animal models, blockade of PD-1 partially reversed T-cell exhaustion, and multiple blockades of inhibitory receptors enhanced T-cell function more efficiently. For example, combined blockade of PD-1 and LAG-3, PD-1 and CTLA-4, and PD-1 and TIM-3 were more efficient. Blockade with monoclonal antibodies targeting the inhibitory receptors CTLA-4, PD-1 and PD-L1 emerged as a successful therapy for patients with advanced melanoma. The durable tumor responses were achieved with PD-1 and PD-L1 blockade in phase I trials in many cancers, and tumor responses were observed in a higher proportion of patients with melanoma than typically observed with ipilimumab, indicating that the blockade of the PD-L1/PD-1 pathway is a more promising strategy for cancer treatment. However, there still exists some limitation in T-cell exhaustion research. Firstly, the different regulation roles of inhibitory receptors remain to be elucidated, for example, PD-1 and TIM-3 may regulate different process of T-cell exhaustion. Secondly, reversing exhausted T cells in cancer may induce excessive T-cell activation and cytotoxicity, leading to adverse reaction, more intervention should be applied to attenuate cytotoxicity-induced injury. Thirdly, benefits from inhibitory receptors blockade are limited, more combined therapies should be applied to enhance response rate. These will advance our fundamental understanding of TME and carcinogenesis. Importantly, the approach of reversing T-cell exhaustion in TME provides a promising avenue to treat cancer.

## Figures and Tables

**Table 1:** The prognostic significance of PD-L1 and PD-L2 in some malignancies

**Cancer**	**Prognostic significance**	**Reference**
Esophageal cancer	PD-L1 and PD-L2 status may be predictor of prognosis for patients with esophageal cancer	^16^
Hepatocellular carcinoma	Patients with higher expression of PD-L1 have a significantly poorer prognosis,patients with higher expression of PD-L2 also have a poorer survival	^17^
Soft tissue sarcoma	PD-L1 expression are significantly associated with advanced clinicopathological parameters, and PD-L1 positivity is independent prognostic indicator of overall survival and event-free survival	^18^
Adrenocortical carcinoma	PD-L1 expression in adrenocortical carcinoma has no relationship to clinicopathologic parameters or survival	^19^
Non-small cell lung cancer	PD-L1 or PD-L2 expression is associated with advanced clinicopathologic features and poor overall survival	^20^
Breast cancer	PD-L1 expression is an independent negative prognostic factor for overall survival	^21^
Ovarian cancer	PD-L1 expression is independent negative prognostic factors, so is PD-L2 expression	^22, 23^
Melanoma	There is a correlation between the degree of PD-L1 expression and the vertical growth of primary tumors in melanoma	^24^
Pancreatic cancer	Combined PD-1/PD-L1 expression can serve as an independent prognostic marker for pancreatic carcinoma, PD-L2 shows no siginicant correlation with patient survival	^25, 26^
Cervical cancer	PD-L1 expression influences patient survival	^27^
Colon cancer	PD-L1 expression is associated with TMN stage and prognosis	^28^

Abbreviations: PD-L1, programmed cell death 1 ligand 1; PD-L2, programmed cell death 1 ligand 2

**Table 2:** Therapeutic interventions for blocking immune checkpoints

**Antibody**	**Target**	**Company**	**Status of clinical development**	**Cancer type**
Ipilimumab	CTLA-4	Bristol-Meyers Squibb	FDA approved for advanced melanoma, phase II and III trial for other cancers	Melanoma, solid tumors
Tremelimumab	CTLA-4	Pfizer	Phase II	Mesothelioma
Nivolumab	PD-1	Bristol-Meyers Squibb	Phases I and II	Solid tumors, melanoma, NSCLC, RCC, ovarian cancer
Pidilizumab	PD-1	CureTech	Phases I and II	Hematologic malignancies
Pembrolizumab	PD-1	Merck	Phase I	Melanoma, NSCLC, head and neck cancer
BMS-936559	PD-L1	Bristol-Meyers Squibb	Phase I	Solid tumors
MPDL3280A	PD-L1	Roche	Phase I	Solid tumors, melanoma, NSCLC, bladder cancer
MEDI4736	PD-L1	MedImmune	Phase I	Solid tumors, melanoma, head and neck cancer, gastric cancer

Abbreviations: CTLA-4, cytotoxic T lymphocyte antigen-4; NSCLC, non-small cell lung cancer; PD-1, programmed cell death protein 1; PD-L1, programmed cell death 1 ligand 1; RCC, renal cell carcinoma

## Abbreviations

AP-1: activator protein 1
BTLA: band T lymphocyte attenuator
CTLA-4: cytotoxic T lymphocyte antigen-4
DC: dendritic cell
GzmB: granzyme B
IDO: indoleamine 2,3-dioxygenase
IFN: interferon
IL: interleukin
LAG-3: lymphocyte activation gene 3 protein
LCMV: lymphocytic choriomeningitis virus
mAb: monoclonal antibody
MDSC: myeloid-derived suppressor cell
NSCLC: non-small cell lung cancer
PD-1: programmed cell death protein 1
PD-L1: programmed cell death 1 ligand 1
PD-L2: programmed cell death 1 ligand 2
RCC: renal cell carcinoma
TAM: tumor-associated macrophage
TCR: T-cell receptor
TGF: transforming growth factor
TIGIT: T-cell immunoglobulin and immunoreceptor tyrosine-based inhibitory motif domain
TIL: tumor-infiltrating lymphocyte
TIM-3: T-cell immunoglobulin domain and mucin domain protein 3
TME: tumor microenvironment
TNF: tumor necrosis factor
Treg: regulatory T cell
